# Serratiopeptidase, A Serine Protease Anti-Inflammatory, Fibrinolytic, and Mucolytic Drug, Can Be a Useful Adjuvant for Management in COVID-19

**DOI:** 10.3389/fphar.2021.603997

**Published:** 2021-06-24

**Authors:** Charu Sharma, Niraj Kumar Jha, M. F. Nagoor Meeran, Chandragouda R. Patil, Sameer N. Goyal, Shreesh Ojha

**Affiliations:** ^1^ Department of Internal Medicine, College of Medicine and Health Sciences, United Arab Emirates University, Al Ain, United Arab Emirates; ^2^ Department of Biotechnology, School of Engineering and Technology (SET), Sharda University, Greater Noida, India; ^3^ Department of Pharmacology and Therapeutics, College of Medicine and Health Sciences, United Arab Emirates University, Al Ain, United Arab Emirates; ^4^ Department of Pharmacology, Delhi Pharmaceutical Sciences and Research University, New Delhi, India; ^5^ Shri Vile Parle Kelavani Mandal’s Institute of Pharmacy, Dhule, India

**Keywords:** COVID-19, infection, inflammation, immunomodulators, serratiopeptidase, drug repurposing

## Introduction

The COVID-19 pandemic, which is caused by severe acute respiratory syndrome coronavirus 2 (SARS-CoV-2), is a public health emergency with high mortality and disability rates. Given its high mortality rate, there is a serious need for possible effective medications to eliminate the virus, limit the severity, and improve the prognosis (Altay et al., 2020). The management of COVID-19 has continued to rely on drugs repurposed based on their pharmacological effects, including antiviral, antibiotic, anti-inflammatory, and or immunomodulatory, along with availability of numerous vaccines against SARS-CoV-2 in past few months (Fan et al., 2020). Repurposing of drugs has gained enormous attention over identifying novel drug candidates, due to known safety, potency, and multi-targeted pharmacological action as an immunomodulatory, anti-inflammatory, and antimicrobial agent. Studies report that after fever, cough is one of the major symptoms in about 76% patients and sputum production in 28% patients along with 55 and 44% of patients showing dyspnea and myalgia, respectively (Huang et al., 2020). In a study determined the prevalence of asymptomatic cases of COVID-19 and characterized the symptoms of patients with mild COVID-19 report that of the 213 individuals with COVID-19, 19.2% were asymptomatic until admission (Kim et al., 2020). Among the remaining patients with mild COVID-19, cough (40.1%) was the most common symptom followed by hyposmia (39.5%) and sputum (39.5%). In individuals with hyposmia, 90% had accompanying symptoms such as hypogeusia, nasal congestion or rhinorrhoea (Kim et al., 2020). Sputum or productive cough seem a significant symptom in asymptomatic as well as symptomatic (Kim et al., 2020). Cough was observed most common symptom followed by hyposmia and sputum, while fever (>37.5°C) was only observed in 11.6% (Kim et al., 2020). Another study reported that nasal congestion (62%) was the most common symptom in individuals with mild COVID-19 (Chang et al., 2020).

The role of mucolytic and bronchodilator administration and tracheal suctioning have been observed beneficial in airway hygiene by reducing the mortality rate of COVID-19 (Farooqi et al., 2020). Therefore, the role of mucolytics, in particular, has been suggested to protect the body from respiratory pathogens ascribed to their expectorant action, and are considered important as an adjuvant in the management of COVID-19 (Esam, 2020). In the purview of the pharmacological basis of therapeutics, we hypothesize that a proteolytic drug of natural origin, serratiopeptidase (SEPD), also known as *Serratia E-15 protease* or serralysin, serratiaprotease and serrapeptase (Bhagat et al., 2013). SEPD (EC number 3.4.24.40), a serine protease super is derived from the non-pathogenic enterobacteria, which exists in the intestine of the silkworm and facilitates disruption of the cocoon to free the silk moth (Maeda and Morihara, 1995). The forms used in pharmaceutical preparations are isolated from *Serratia marcescens* or *Serratia* sp*. E 15* based on fermentation or the recombinant production using *Escherichia coli* (Srivastava et al., 2019)*.*

Enzyme drugs are reputed in therapeutics due to their strong target binding and specificity and catalytic behavior to change many target molecules into the desired effectors (Reshma, 2019). Proteolytic enzymes can be useful in the treatment of nosocomial, viral, and resistant infections, especially in pediatric and geriatric age groups due to its relative safety, less tolerance and resistance and its synergic effects (UmaMaheswari et al., 2016). Several proteolytic enzymes act in an orchestrated manner to control and coordinate the entry of virus, replication and diffusion in the host cells. Thus, the proteolytic enzymes could be important in interfering with virus machinery in the host cells and suggested useful in COVID-19 (Gioia et al., 2020). Recently, SEPD has been suggested to be considered in integrative management of COVID-19 (Holloway et al., 2020). One of the case report suggested the role of immunostimulants and proteolytic including SEPD in the treatment of COVID-19 (Kobakova et al., 2020).

Our proposition is to repurpose a drug that possesses not only mucolytic property but also potent anti-inflammatory, and antimicrobial properties with a long history of safe clinical use. Herein, we present the possibilities of repurposing SEPD, a mucolytic that could be advantageous over others in COVID-19 treatment due to its wide range of therapeutic effects, including anti-inflammatory, antimicrobial, atheroprotective, antithrombotic, and fibrinolytic properties. Based on these properties, we opined that these properties may provide better therapeutic benefits in limiting the severity and progression of the disease, by reducing the risks of respiratory complications and related death.

### Serratiopeptidase as A Mucolytic Drug Can Be Useful in COVID-19

In individuals with COVID-19, sputum production, nasal congestion and cough are reported one of the common symptoms after fever (Chang et al., 2020; Huang et al., 2020; Kim et al., 2020). As cough is a major symptom of SARS-CoV-2 infection, the caseinolytic and mucolytic actions of SEPD on the sputum believed to be beneficial. Recently, one of the mucolytic drugs, bromhexine, has been suggested to be repurposed for the possible treatment of COVID-19 (Maggio and Corsini, 2020). Mucolytics either enhance bronchial mucus secretion or reduce mucus viscosity and further facilitate its removal by coughing. The mucus secreted by the goblet cells is an adhesive viscoelastic gel containing high molecular weight mucous glycoproteins and water. The airway mucus is well-known as the first line of airway defense against pathogens, including coronaviruses. The hypersecretion of the airways mucus in a defensive response to the pathogens are believed to cause airway obstruction that leads to respiratory distress (Lu et al., 2021).

The mucus in airways traps and keep the microorganisms by a coordinated process of mucociliary clearance which involves release of mucus from the secretory cells controlling the transportation and viscoelasticity by motile cilia on multiciliated cells (Janssen et al., 2016). Mucus accumulation and increase in sputum viscoelasticity reduce mucociliary and cough clearance, thus retaining the sputum and obstructing the airways that enhance inflammation, infection, and progressive lung diseases by neutrophil infiltration (Maggio and Corsini, 2020). SEPD is shown to enhance mucociliary transportability (Maheshwari et al., 2006) and mucociliary clearance by decreasing neutrophils and modulating sputum viscoelasticity in patients with airway diseases (Nakamura et al., 2003). In addition to the mucolytic property, SEPD through oral administration in allergic conditions decreases the viscosity of the nasal mucus by improving rheological properties; thus, it plays a role in mucociliary clearance (Majima et al., 1988; Majima et al., 1990). SEPD has been found bioavailable in the nasal or tracheobronchial mucus, and it exerts proteolytic action even after oral intake (Majima et al., 1988; Majima et al., 1990).

Recently, the role of mucins glycoproteins, the structural components of mucus and its interaction with microorganisms particularly SARS-CoV-2 and its pathophysiological and therapeutic relevance has been presented to enhance mucosal defense and control respiratory infections (Chatterjee et al., 2020). The elevated levels of mucin has been reported in the airway mucus of critical ill COVID-19 patients (Lu et al., 2021). The higher levels of mucins are reported in the COVID-19 patients bronchoalveolar lavage fluid (BALF) and lungs of preclinical models of SARS-CoV-2 (Liu et al., 2020). Liu et al. (2020) suggested that during SARS-CoV-2 infection, the rise in the IFN-β and -γ leads higher expression of mucins in alveolar epithelial cells. The mucins stick with the blood-gas barrier and accumulated alveolar mucus affects the blood-gas barrier thereby impeding the gaseous exchange of O_2_ and CO_2_ and causing hypoxia, a key factor that initiates COVID-19-induced mortality. Following progression in the diseases, increase in barrier thickness, along with raised inflammatory exudates causes impediment in exchange of O_2_ and CO_2_ that leads to the critical illness and complications (Liu et al., 2020).

Additionally, SEPD has shown useful in chronic respiratory diseases (Nakamura et al., 2003), chronic sinusitis (Majima et al., 1988), ear, nose and throat disorders (Mazzone et al., 1990), secretory otitis media (Bellussi et al., 1984) and chronic airway disease with troubled expectoration (Nagaoka et al., 1979). Based on the role of SEPD on mucociliary clearance, relieving cough and promoting airway hygiene, it may be useful in delaying pulmonary complications and improving quality of life in COVID-19.

### Serratiopeptidase as an Anti-inflammatory Drug Can Be Useful in COVID-19

The anti-inflammatory effects of SEPD were reported in the late 1960s, and since then, it has been popularly used in therapeutics for inflammatory diseases in Japan and many European and Asian countries (Gupte and Luthra, 2017; Tiwari, 2017; Jadhav et al., 2020). Currently, it is available in United States, Canada and European countries as a natural health supplement or dietary ingredient, rather as a drug (Jadhav et al., 2020). It has been widely used in the management of pain and inflammation related to joints, sports-related chronic muscular swelling, sprain, scar, ruptured ligaments, chronic swelling and injuries, sinusitis, bronchitis, carpel tunnel syndrome, tooth extraction, breast engorgement, and post-surgery inflammation (Mazzone et al., 1990; Klein and Kullich, 2000; Tiwari, 2017).

SEPD has been shown to exert anti-inflammatory effects by reducing inflammatory cytokines and adhesion molecules, thus regulate inflammatory cells movement to the site of inflammation (Tiwari, 2017). It has been reported safer than conventional nonsteroidal anti-inflammatory drugs in terms of safety and efficacy and showed synergistic with them as well as with metal ions like zinc and manganese (Tiwari, 2017). SEPD has been shown to exert anti-inflammatory, antiedemic and fibrinolytic activity in resolving inflammation in patients with acute or chronic ear, nose or throat disorders in a multicenter, double blind, placebo-controlled study (Mazzone et al., 1990).

SEPD has been demonstrated to reduce neutrophil count and altering the viscoelasticity of sputum in patients with airway diseases (Nakamura et al., 2003). A reduction in the neutrophil count is believed to reduce elastase, a serine protease released from activated neutrophils in host defense response to attack proteins of pathogens; facilitate protein hydrolyzation in the host extracellular matrix, particularly collagen IV and elastin; ensue inflammation; and increase virus multiplication (Thierry, 2020). Elastase in the lungs can cause excessive water absorption that dehydrates the mucus and causes inefficient mucociliary clearance. Elastase also promotes the generation of ROS, alters the permeability of lung barriers, and triggers pro-inflammatory cytokines. Thus, elastase inhibition by SEPD could be useful in suppressing cytokine storm, causing acute lung injury in COVID-19. Inhibition of elastase by SEPD in the airways may also suppress airway inflammation characterized by reduced bronchial injury, improved ciliary beating, and reduced mucus hypersecretion (Thierry, 2020).

Additionally, the elevated levels of inflammatory cytokines, including interleukin (IL)-6 play vital role in pathogenesis and progression of complications, severity and mortality in COVID- 19 (Cummings et al., 2020; Hojyo et al., 2020; Wang J. et al., 2020). The clinical manifestations of COVID-19 can range from mild to severe with widespread involvement of the lungs, beginning from pneumonia to acute respiratory distress, involving extensive alveolar damage along with progressive lung dysfunction, and leading to respiratory failure that may result in death (Yang et al., 2020).

Acute respiratory distress, which cause acute lung injuries characterized by infiltration of neutrophils, vasculitis, and secretion of proinflammatory cytokines, particularly results in a massive increase in IL-6 level, which has been found to be related to the severity of the disease, prognosis, and mortality (Giamarellos-Bourboulis et al., 2020; Han et al., 2020). Increased IL-6 levels also contribute to acute lung injury in murine models (Goldman et al., 2014), similar to those observed in patients with severe acute respiratory syndrome in COVID-19; thus, inhibition of enhanced IL-6 level seems to mitigate acute lung injury (Goldman et al., 2014; Pelaia et al., 2020). In a recent study, SEPD and curcumin nanoparticles (NPs) are shown to exert potent IL-6 inhibitory activity as evidenced by the reduction in IL-6 level ranging from 47 to 80% in lipopolysaccharide-stimulated human macrophages (Jaiswal and Mishra, 2018). The NPs of SEPD and curcumin showed potent synergetic immunomodulatory and anti-inflammatory properties (Jaiswal and Mishra, 2018). SEPD also found to inhibit IL-6, transforming growth factor-β (TGF-β) expression, chemokines (Selan et al., 2017), in the brain tissues of rat model of aluminum chloride-induced Alzheimer’s disease (Fadl et al., 2013) and blood (Iie, 2013) after oral administration. SEPD has been demonstrated to attenuate proinflammatory cytokines in pulmonary tissues following liposomal delivery (Gupta et al., 2017).

Furthermore, the hyperinflammatory responses also involved the overproduction of bradykinins, which in turn determine disease severity, progression and mortality (Henderson et al., 2020). Bradykinin is one of the potent components of the vasopressor system that is degraded by angiotensin converting enzyme (ACE) and upon induction causes hypotension, vasodilation and natriuresis (Hofman et al., 2016). The increased bradykinin level from serine protease kallikrein has been determined to contribute to vasodilation, hypotension, and altered vascular permeability and can further lead to excessive formation of hyaluronic acid in the bronchoalveolar space of the lungs, which impairs lung function and plays a role in the onset of inflammation and pain (Garvin et al., 2020). The downregulation of the enzymes which degrade bradykinin are reported in bronchoalveolar lavage fluid (BALF) of patients with severe/critical COVID-19 infection (Garvin et al., 2020). The decrease in the enzymes is believed to shift the renin angiotensin system to produce Ang mediating ACE2. The upregulation of ACE2 and reduced degradation of bradykinin by ACE is believed to cause “bradykinin storm” which induces leakage of fluid into the lungs and it combines with hyaluronic acid forms a Jello-like material. This sticky formation obstructs exchange of O_2_ and CO_2_ and leads to the severe complications in COVID-19 (Garvin et al., 2020)^.^

Additionally, SEPD has been showed to exert anti-inflammatory effects by inhibiting the release of serotonin and histamine. The anti-inflammatory activity of SEPD at the systemic and cellular level is suggestive of its potential in limiting cellular injury in different organs by inhibiting inflammation. Therefore, it can be suggested that SEPD may reduce acute respiratory distress and limit complications in COVID-19 ascribed to its inhibitory effect on bradykinin, serotonin, and histamine (Malshe, 2000).

### Serratiopeptidase Potential in Coagulopathy and Thrombosis Complications

In addition to inflammatory cytokines, higher bradykinin levels with increased growth factor levels exhibit a strong association between inflammation and coagulation (Hofman et al., 2016). Further, histamine and bradykinin, the vasoactive mediators are implicated in mucosal swelling. The neutrophil and mast-cell activation along with fibrinolytic system activation (i.e. plasminogen activation) are functionally linked to bradykinin production and considered to play role as one of important inflammatory product of the coagulation system (Hofman et al., 2016). Higher fibrinogen and lower antithrombin levels were reported in patients with COVID-19 and associated with the severity of infection, mortality, and prognosis in survivors (Tang et al., 2020). The development of thrombosis characterized by a significant increase in D-dimer and fibrin/fibrinogen-degradation products with coagulopathy is one of the major causes of cardiovascular complications in patients with COVID-19 (Connors and Levy, 2020).

Additionally, enhanced degradation products of fibrin have been identified to play a role in intravascular coagulation, a manifesation of viral coagulopathy following arterial, venous, and microvascular thrombosis and endothelial damage in the lungs that leads to acute respiratory distress syndrome (ARDS) (Kipshidze et al., 2020).

Many fibrinolytic therapies and tissue plasminogen activators based on serine protease known for their benefits in vascular disorders have been suggested to aid in COVID-19 treatment (Lechowicz et al., 2020). SEPD has been reported to holds extensive substrate affinity and fibrinolytic property (Kotb, 2013). SEPD possesses the ability to degrade blood clots, cysts, and arterial plaques, therefore being useful under the conditions of increased risk of stroke, atherosclerosis, and thrombophlebitis (Mazzone et al., 1990). The fibrinolytic activity of SEPD coupled with multiple properties, including proteolytic, caseinolytic, antifibrotic, anti-inflammatory, antiatherosclerotic, and antioxidant activity, suggests its potential benefits in reducing the severity of vascular complications involving thrombosis or coagulopathy in COVID-19.

### Serratiopeptidase Potential in Countering Oxidative Stress

The extrapulmonary complications of COVID-19 are acute liver injury, acute cardiac injury, acute intestinal inflammation, and acute neurological manifestations, which may further lead to sepsis and multi-organ failure with poor prognosis (Wang et al., 2020; Xu et al., 2020). The pathogenesis of acute complications of different organs involves an abrupt disruption in antioxidant defense against oxidative stress subsequent to systemic hyperinflammatory response (Henderson et al., 2020). Further, serine protease enzymes showed to exert free radical scavenging activity that also help in its therapeutic benefits (Davies, 1986). SEPD conjugated with folate and superoxide dismutase has been considered useful in inflammatory conditions by enhancing retention and localized delivery of the conjugate along with augmentation of proteolytic activity and free radical scavenging activity against reactive oxygen species (ROS) generated from macrophages (Srivastava et al., 2017). Thus, the antioxidant activity may also contribute to tissue protective effects and explain therapeutic benefits of SEPD in reducing organ complications.

### Serratiopeptidase Synergizes Antibacterial Drugs and Corticosteroids

In COVID-19, the increased risk of secondary bacterial infections in critically ill patients contribute to the cumulative inflammatory burden in addition to viral pneumonia and has been reported to cause complications and death (Fu et al., 2020). SEPD exerts synergistic antimicrobial activity with drugs belong to the antibiotic family of penicillins, cephalosporins, fluoroquinolones, and tetracyclines (Maheshwari et al., 2006). SEPD was found to eradicate implant related periprosthetic infection in an *in vivo* animal model of staphylococcal infections (Mecikoglu et al., 2006). It has also been showed a valuable agent in combination with antibiotics and anti-inflammatory agents in the treatment of periimplantitis (Sannino et al., 2013).

SEPD has also been shown to enhance the absorption of antibiotics and prevent biofilm formation in pulmonary tissues in patients undergoing thoracotomy (Koyama et al., 1986). The pulmonary delivery of SEPD with levofloxacin in liposomes exerts potent antimicrobial activity against *Staphylococcus aureus* infections in rats and reduces bacterial resistance by inhibiting biofilm formation. This combination was found bioavailable and synergistically effective in respiratory infections and has further reduced the doses of levofloxacin for bacterial infections (Gupta et al., 2017). SEPD in preclinical studies showed to increase the levels of cefotiam in plasma and lungs in pleuritis and only in lungs in pneumonitis (Ishihara et al., 1983), in subacute bronchitis (Kase et al., 1982) and synergizes the efficacy of ciclacillin, ampicillin, cephalexin and minocycline in gingival infections caused by staphylococci (Aratani et al., 1980).

Additionally, SEPD has been reported to synergize corticosteroid drugs methylprednisolone and dexamethasone (Murugesan et al., 2012), which received attention for their potential use in COVID-19 (Tomazini et al., 2020). In acute respiratory distress, corticosteroids, mainly methylprednisolone, improve oxygenation, lessen the requirement of mechanical ventilation, and decrease mortality risks (Steinberg et al., 2006). However, high doses or prolonged use of corticosteroids may result in excessive immune suppression and related mortality. Hence, when the pathogenesis progresses from inflammation to fibrosis, the adverse effects of anti-inflammatory drugs likely outweigh any potential benefit. SEPD does not directly interfere with lipoxygenase enzymes, which are a major target of non-steroidal anti-inflammatory drugs (NSAIDs), therefore being devoid of numerous adverse effects and exhibiting synergistic effect in combination with NSAIDs. The synergistic and comparable action of SEPD with methylprednisolone and dexamethasone is suggestive of its potential in limiting respiratory distress and delaying the requirements of mechanical ventilation (Murugesan et al., 2012).

### Serratiopeptidase May Be Useful in Pulmonary Fibrosis in COVID-19

There are reports that in some COVID-19 survivors, pulmonary fibrosis develops as a post-infection sequela (Lechowicz et al., 2020). Pulmonary fibrosis is often characterized by activation of TGF-β and matrix metalloproteinase, fibroblast proliferation mediated by accumulation of collagen and extracellular matrix, and injury to alveolar epithelium and parenchyma and capillaries that may lead to difficulty in breathing and may cause acute respiratory failure (MacLaren and Stringer, 2007). TGF-β1 is one of the major contributors to fibrosis and ROS production. Excessive production of ROS that induces oxidative stress and overexpression of cytokines contributes to pulmonary fibrosis. The proteolytic activities are considered as a secondary antioxidant defense in oxidative conditions, along with regulation of inflammatory cytokines and migration of immune cells from the lymph node to the inflamed and injured tissues (Tiwari, 2017). The ability of SEPD to suppress growth factors, particularly TGF-β along with inhibiting oxidative stress and expression of pro-inflammatory cytokines, chemokines, adhesion molecules (Fadl et al., 2013; Gupta et al., 2017; Jaiswal and Mishra, 2018), plausibly indicates its possible potential in the treatment of lung fibrosis.

### Serratiopeptidase Doses, Safety, and Adverse Effects

SEPD is generally well tolerated with few exceptions of rare adverse effects. It is available alone or in combination with anti-inflammatory agents as tablet, mostly as enteric-coated tablets or capsule. SEPD is distributed to the tissues and bioavailable in plasma and lymph following binding to alpha-2-macroglobulin in the blood thus devoid of allergenicity and retains its enzymatic activity at the systemic and cellular level within 1 h.

**FIGURE 1:**
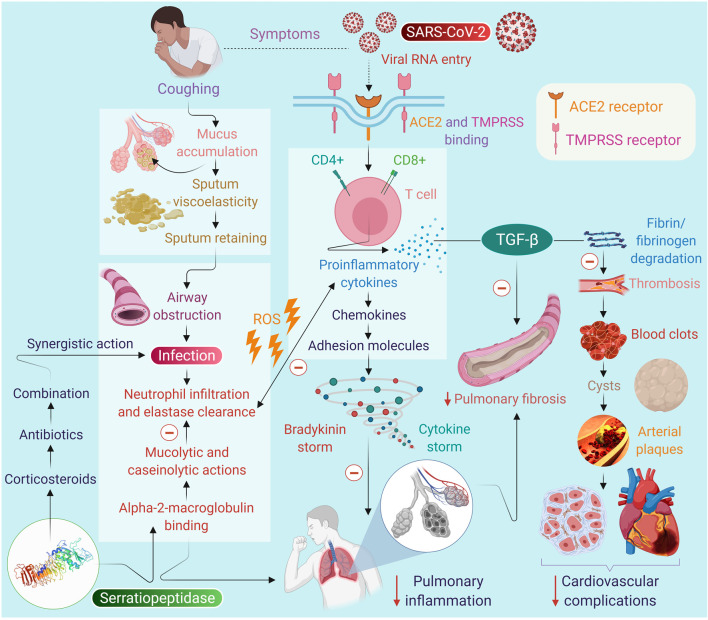
The proposed scheme on the potential of serratiopeptidase on infection, immunity and inflammation in context to SARS-CoV-2 and on the symptoms of COVID-19.

The usual doses of SEPD in a majority of the human studies range from 10 to 60 mg/day in divided doses, with the most preferred dose of 10 mg, thrice daily on an empty stomach. Usually, it is used for 2–4 weeks depending on the aim of therapy and outcome. The dose of 10 mg is considered equal to 20,000 units of enzyme activity. Therefore, we propose that the dose of 10 mg thrice daily could be examined as an adjuvant in COVID-19. Using SEPD can be virtuously justified, being safe and effective and devoid of side effects that commonly develop with the use of conventional mucolytics that may cause sedation, euphoria, gastrointestinal disturbances, respiratory irritation, and constipation probably due to the absence of any interaction with receptors. A scheme is presented in Figure 1 to depict the possible mechanisms and effect of SEPD on mucus production, infection, inflammation, and immunity in the context of SARS-CoV-2.

## Conclusion

SEPD may be a promising therapeutic candidate for repurposing due to its immunomodulatory, anti-inflammatory, mucolytic, antifibrotic, antithrombotic, antiviral, and fibrinolytic properties. SEPD, being an age-old, inexpensive, natural, and tolerated drug, may be a better alternative over other mucolytics or adjuvant with other drugs particularly in individuals with symptoms of sputum or mucus or productive cough. Recently, the animal models of COVID-19 become available that may facilitate preclinical evaluations to distinguish whether these candidate compounds are likely to become effective drugs. Though, the suggestion on the use in COVID-19 remains inconclusive until the proof of concept preclinical and clinical studies undertaken. But the potential of SEPD can’t be overlooked ascribed to its promising possible benefits in COVID-19. It may be able to limit fatal complications, including pulmonary and cardiovascular diseases, and improve the prognosis of COVID-19. However, it is important to highlight that, to date, no studies have demonstrated the experimental or clinical effects of SEPD in COVID-19.
